# Metabolomic Profiling for Histologically Fibrotic Stage in Chronic Drug-Induced Liver Injury

**DOI:** 10.3389/fphar.2022.896198

**Published:** 2022-05-20

**Authors:** Xian He, Ming-Xi Zhou, Cheng Cheng, Shan-Shan Li, Yuan Gao, Zhi-Tao Ma, Xin-Hua Song, Zhao-Fang Bai, Zheng-Sheng Zou, Xiao-He Xiao, Jia-Bo Wang, Ya-Wen Lu

**Affiliations:** ^1^ School of Pharmacy and Chemistry, Dali University, Dali, China; ^2^ School of Traditional Chinese Medicine, Capital Medical University, Beijing, China; ^3^ College of Traditional Chinese Medicine and Food Engineering, Shanxi University of Traditional Chinese Medicine, Shanxi, China; ^4^ Department of Hepatology, Fifth Medical Center of Chinese PLA General Hospital, Beijing, China

**Keywords:** drug-induced liver disease, liver fibrosis, metabolomics, fingerprint, biomarkers

## Abstract

**Background and aims:** Chronic drug-induced liver injury (DILI) is a rare but under-researched adverse drug reaction–related disease, which is highly likely to progress into liver fibrosis and even cirrhosis. In this study, metabolomics was used to screen out characteristic metabolites related to the histological progression of fibrosis in chronic DILI and analyze the metabolic changes during the development of fibrosis to explain the underlying mechanism.

**Methods:** Chronic DILI patients who underwent liver biopsy were divided into different fibrosis grades. Serum was analyzed by untargeted metabolomics to find serological characteristic metabolite fingerprints. The screened fingerprints were validated by the validation group patients, and the identification ability of fingerprints was compared using FibroScan.

**Results:** A total of 31 metabolites associated with fibrosis and 11 metabolites associated with advanced fibrosis were identified. The validation group confirmed the accuracy of the two metabolite fingerprints [area under the curve (AUC) value 0.753 and 0.944]. In addition, the fingerprints showed the ability to distinguish the grades of fibrosis by comparing using FibroScan. The metabolite fingerprint pathway showed that bile acid synthesis is disturbed while lipid metabolism is extremely active, resulting in an overload of lipid metabolites in the occurrence and development of chronic DILI–associated fibrosis.

**Conclusions:** Our metabolomic analysis reveals the unique metabolomic fingerprints associated with chronic DILI fibrosis, which have potential clinical diagnostic and prognostic significances. The metabolomic fingerprints suggest the disturbance of the lipid metabolites as the most important factor in the development of DILI fibrosis.

## 1 Introduction

Drug-induced liver injury (DILI) is a common unexpected adverse drug reaction. Without effective management, individuals with DILI often develop chronic drug-induced liver injury which has a high risk of progressing to liver cirrhosis, hepatocellular carcinoma, and even death. Thus, an accurate diagnosis and prompt blocking disease progression are essential and important in DILI management. However, DILI’s clinical phenotype and etiology is extremely variable, and there no specific biomarkers for DILI’s diagnosis. Hence, diagnosing DILI confidently is extremely difficult.

Although DILI’s etiology is unknown, the clinical progression is clear. The initial stage is the liver inflammation stage, progressing to the hepatic fibrosis stage, and ultimately hepatic cirrhosis or hepatocellular carcinoma, some of which may progress to decompensation of cirrhosis and development of liver failure, and even death. As liver fibrosis is the last reversible course of disease progression, it is necessary to explore the mechanism of fibrosis in DILI patients and detect and intervene in the fibrosis stage of DILI in a timely manner. If the molecular mechanisms of DILI progression could be deciphered, its associated mortality might be reduced.

Metabolites play important roles in intra-organ and inter-organ communications and cellular processes, which could be reflective of the holistic metabolism of the organism. Metabolomics as an emerging field in systems biology focuses on the small-molecule metabolite profiles, and metabolomic analysis has allowed us to gain a novel understanding of metabolism for various states. Several studies have shown that untargeted metabolomics is reliable in screening biomarkers associated with diseases. With numerous success stories demonstrating, metabolomics is an exciting and evolving research area, and its power extends from biomarker discovery to understanding the mechanisms that underlie phenotypes, for example, intense systemic inflammation is associated with blood metabolite accumulation and profound alterations in major metabolic pathways, which may contribute to the development of organ failures in acute-on-chronic liver failure (Johnson et al., 2016; Moreau et al., 2020; Huang et al., 2021a).

Therefore, we collected a prospective cohort of chronic DILI patients. Then, we performed untargeted metabolomics analysis on serum from chronic DILI patients who were grouped by the histopathology of liver biopsies as the reference standard to characterize the serum metabolite fingerprints of DILI-related fibrosis to explore its potential biomarkers and mechanism of development.

## 2 Materials and Methods

### 2.1 Patient Population

The patients included in our study were from the Fifth Medical Center of Chinese PLA General Hospital between November 2015 and May 2017. A total of 96 patients were diagnosed with chronic DILI according to DILI guidelines (Chalasani et al., 2014) and Chinese guidelines on the management of liver cirrhosis (Chinese Society of Hepato, 2019), with no age and sex restriction. A total of 48 hospitalized chronic DILI patients who underwent histopathological examination of liver biopsy from November 2015 to February 2016 were enrolled as discovery phases. The pathological diagnosis of liver fibrosis adopts the Scheuer scoring system to divide patients into three stages: nonsignificant fibrosis group (NF, Scheuer scored S0-1, *n* = 16), significant fibrosis group (SF, Scheuer ≥ S2, *n* = 23, patients in this group showed septal or bridging fibrosis), and advanced fibrosis group (AF, Scheuer ≥ S3, *n* = 9) (Scheuer, 1991). The detailed histopathological diagnosis in patients is shown in Supplementary Table S1. At the same time, 26 of the 48 patients enrolled underwent FibroScan. Another 48 patients from May 2016 to April 2017 formed validation phase groups. In addition, 11 healthy volunteers without age and sex restriction were included as a control group.

This research scheme was approved by the Ethics Committee of the hospital based on the ethical principles of the declaration of Helsinki. All the patients provided their written informed consent to participate in this study.

### 2.2 Sample Preparation and LC/Mass Spectrometry Conditions

Serum samples were processed by the organic solvent precipitation method (Zhang et al., 2020). In brief, 200 µl of a homogenous serum sample collected from patients was mixed with 600 µl precooled methanol. The mixture was vortexed for 30 s and incubated for 20 min at 4°C to precipitate protein and other solid substances. After centrifugation at 14,000 rpm at 4°C for 10 min, the supernatant (400 µl) was transferred to a fresh tube and lyophilized under vacuum. The dried sample were reconstituted in 200 µl 75% methanol and incubated at 4°C for 15 min. The samples were centrifuged at 14, 000 rpm at 4°C for 10 min. Finally, 4 µl of the supernatant was used for LC–MS/MS analysis.

A ZORBOX RRHD C18 column (2.1 × 100 mm, 1.8 μm, Agilent Technologies, CA) and an Agilent 1290 system (Agilent Technologies, CA) equipped with an online degasser, a quaternary pump, an autosampler, and a column compartment were used for chromatographic separation. The column temperature was 30°C and sample injector temperature was 4°C. A gradient elution program was conducted for chromatographic separation with mobile phase A: water (containing 0.1% formic acid and 5% acetonitrile) and mobile phase B: acetonitrile (containing 0.1% formic acid) as shown in Supplementary Table S2A. The flow rate was 0.30 ml/min. An Agilent 6550 iFunnel Q-TOF LC/MS equipped with electron spray ionization (ESI) was used for mass spectrometry (MS) evaluations. MS data were acquired in both positive and negative modes based on optimal parameters shown in Supplementary Table S2B.

All mobile phases were freshly prepared, and the sequence order of samples was randomized. For data quality assessment, the quality control (QC) samples were prepared by mixed samples.

### 2.3 Statistical Analysis

The raw data were transformed and preprocessed using Agilent MassHunter Profinder, and the relative ion intensity was normalized to the sum of the peak area. The *p*-values, fold change (FC), and the area under the receiver operating characteristic curve (AUC) were obtained from the online analysis tool MetaboAnalyst 5.0 (https://www.metaboanalyst.ca). Differential metabolites were screened according to the conditions (*p* < 0.05, FC > 1.5 or <0.67). The metabolites were identified in the Human Metabolome Database (HMDB, http://www.hmdb.ca) based on the m/z value and retention time. Statistical analysis was performed using SPSS 25.0 software (Chicago, United States). The statistical significance was analyzed using the unpaired two-tailed Student’s t test, and *p* < 0.05 was considered statistically significant. Features such as statistical significance were imported into the SIMCA-P program (version 14.1, Umetrics) for principal component analysis (PCA) and orthogonal partial least-square discrimination analysis (OPLS-DA). GraphPad Prism 8.0 version was used to draw heat maps, the receiver operating characteristic (ROC) curve, and binary logistic regression. Kyoto Encyclopedia of Genes and Genomes (https://www.kegg.jp) was used to construct an interactive network for the metabolite fingerprint.

## 3 Results

### 3.1 Baseline Demographics and Clinical Characteristics of the Study Cohort

The discovery group included 48 participants. The detailed demographic and baseline laboratory data are shown in Table 1. There was no significant difference in the average age between the two groups, but there was female predominance (*n* = 35, 72.9%) in this study. There were no statistical differences in other serological parameters between the groups, except the level of albumin, which significantly decreased in the fibrosis group (SF, AF group) compared with the NF group. Albumin deficiency is very common in patients with liver disease, indicating impaired liver function. The validation group also included 48 participants, and the demographic information is shown in Supplementary Table S3.

**TABLE 1:** Clinical characteristics of the study patients in NF, SF, and AF groups.

Characteristics	NF (*n* = 16)	SF (*n* = 23)	AF (*n* = 9)	*p*-value		
				NF vs. SF	NF vs. AF	SF vs. AF
Age/year	47.0 (41.5–51.8)	52.2 (46.0–55.2)	52.1 (48.0–57.0)	0.267	0.156	0.579
Female, *n* (%)	10 (62.5)	17 (73.9)	8 (88.9)	-	-	-
ALT/U L^−1^	69.0 (18.8,126.8)	159.0 (55.0–298.5)	143.0 (78.0–173.0)	0.273	0.877	0.236
AST/U L^−1^	25.0 (19.0–170.3)	111.0 (72.0–290.5)	132.0 (101.0–267.0)	0.124	0.085	0.743
AST/ALT	1.0 (0.6–1.4)	0.7 (0.5–1.9)	1.4 (0.7–2.2)	0.296	0.155	0.567
ALP/U L^−1^	104.0 (65.0–127.0)	104.0 (85.5,135.5)	108.0 (102.0–123.0)	0.853	0.461	0.375
GGT/U L^−1^	118.0 (35.8,161.8)	83.0 (54.5,156.5)	148.0 (102.0–240.0)	0.396	0.734	0.145
ALB/g L^−1^	39.0 (37.5–40.3)	36.0 (34.0–37.5)	35.0 (35.0–35.0)	0.011	0.001	0.070
TBIL/µmo L^−1^	12.0 (9.6–18.8)	14.3 (9.2–37.4)	49.3 (27.0–53.3)	0.622	0.657	0.908
DBIL/µmo L^−1^	4.6 (3.6–7.6)	8.7 (3.9–30.3)	35.5 (8.5–43.7)	0.554	0.562	0.954
CHE/U L^−1^	6978.5 (5407.8–7850.8)	5511.0 (4502.5–6957.5)	5550.0 (4220.0–6134.0)	0.205	0.052	0.542
TC/mmol L^−1^	4.5 (3.7–5.3)	3.6 (3.2–4.7)	4.0 (2.9–4.4)	0.172	0.191	0.956
TG/mmol L^−1^	1.4 (1.2–1.8)	1.6 (0.9–2.1)	1.6 (1.3–2.3)	0.866	0.268	0.159
IgA/g L^−1^	2.2 (1.4–2.4)	2.1 (1.7–2.8)	2.0 (1.6–3.2)	0.324	0.375	0.772
IgG/g L^−1^	12.6 (8.9–15.8)	14.8 (12.5–18.2)	12.6 (11.7–14.7)	0.108	0.825	0.289
IgM/g L^−1^	0.9 (0.7–1.3)	1.1 (0.7–1.2)	0.9 (0.5–1.0)	0.835	0.525	0.537
INR/IU	1.0 (0.9–1.0)	1.0 (0.9–1.1)	1.0 (0.9–1.1)	0.170	0.112	0.490
CRE/µmo L^−1^	66.0 (56.0–75.8)	60.0 (51.0–67.5)	58.0 (52.0–66.0)	0.081	0.326	0.894
PT/s	11.0 (10.7–11.4)	11.1 (10.6–12.2)	11.6 (10.8–12.8)	0.135	0.103	0.504

ALT, alanine aminotransferase; AST, aspartate transaminase; ALP, alkaline phosphatase; GGT, gamma-glutamyl transpeptidase; ALB, albumin; TBIL, total bilirubin; DBIL, direct bilirubin; CHE, cholinesterase; TC, total cholesterol; TG, triglyceride; INR, International standard ratio; CRE, creatinine; PT, prothrombin time. NF, nonsignificant fibrosis; SF, significant fibrosis; AF, advanced fibrosis.

Data are median (p25, p75) or numerical value. *p*-values for comparisons were carried out by nonparametric tests.

### 3.2 Serum Metabolite Profile Related to Fibrosis in Patients With Chronic Drug-Induced Liver Injury

**FIGURE 1:**
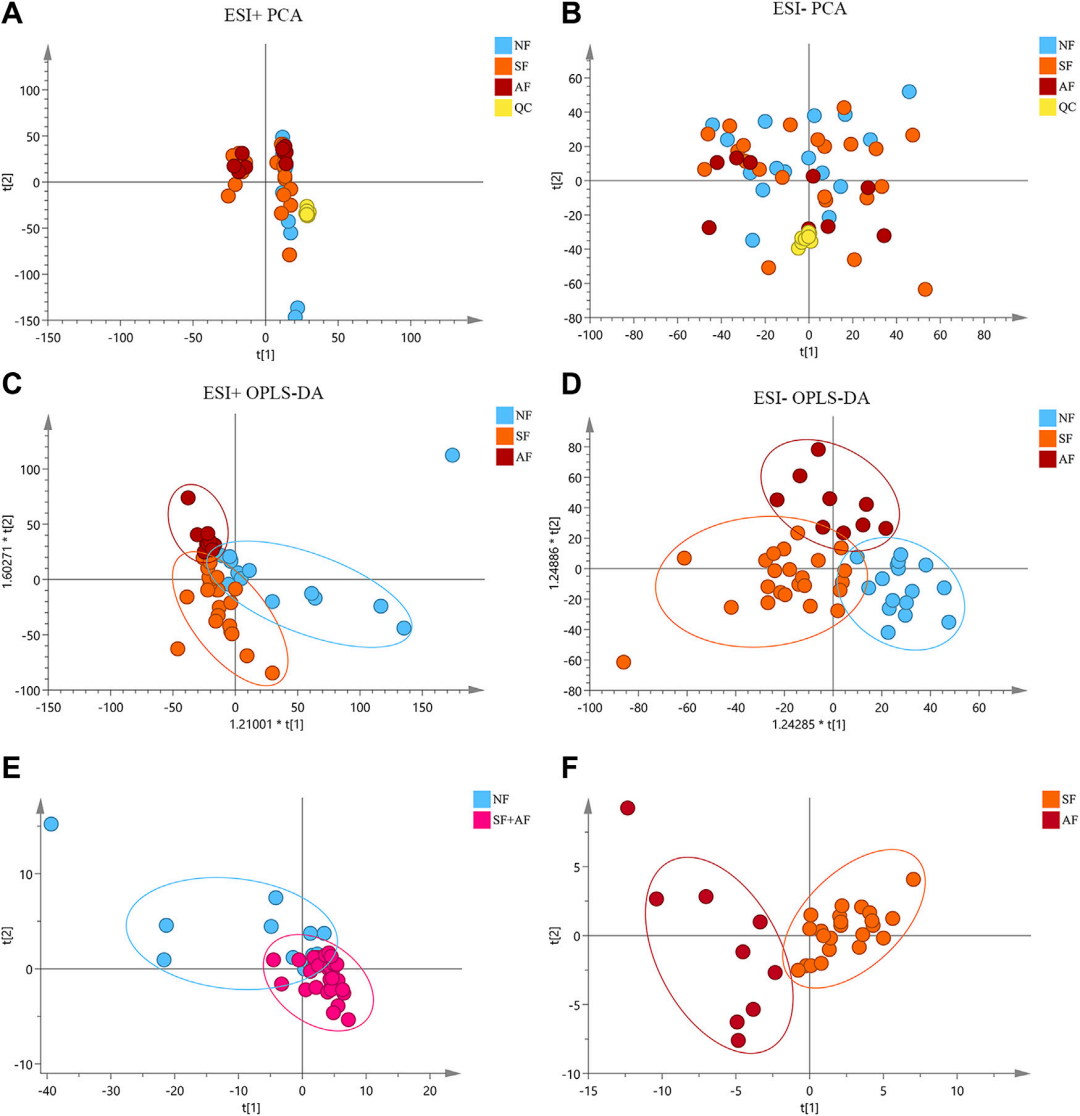
Serum metabolite profile related to fibrosis in patients with chronic DILI. **(A,B)** Principal components analysis (PCA) of all variables among the NF, SF, and AF groups under the positive and negative models of mass spectrometry. **(C,D)** Orthogonal projections to latent structures discriminant analysis (OPLS-DA) of all variables among NF, SF, and AF groups under the positive and negative models of mass spectrometry. **(E)** PCA plot of (SF + AF) vs. NF with 273 annotated metabolites related to fibrosis. **(F)** PCA plot of SF vs. AF with 96 annotated metabolites related to advanced fibrosis. NF, nonsignificant fibrosis; SF, significant fibrosis; AF, advanced fibrosis.

Significant changes were observed in the metabolite profile of chronic DILI patients in both positive and negative modes of mass spectrometry (Figures 1A–D), which suggested that metabolism had changed in liver fibrosis progresses. It was possible to identify different stages of fibrosis using differential metabolites as fingerprints. Following the criterion shown in patients and methods, 273 metabolites were selected (*p* < 0.05, FC >1.5 or <0.67) and identified by comparing the correct LC/MS fragments in the Human Metabolome Database, excluding interference of annotations, environmental pollutants, and characteristic metabolites of drugs taken. These metabolites were considered to be associated with significant fibrosis, which could distinguish the NF group from the fibrosis group (Figure 1E). Using the same approach, we compared the SF with AF groups and identified 96 differential metabolites associated with advanced fibrosis that distinguished the SF group from the AF group (Figure 1F).

### 3.3 Serum Metabolites Compose Fingerprints Related to Progressions of Liver Fibrosis in Chronic Drug-Induced Liver Injury Patients

**FIGURE 2:**
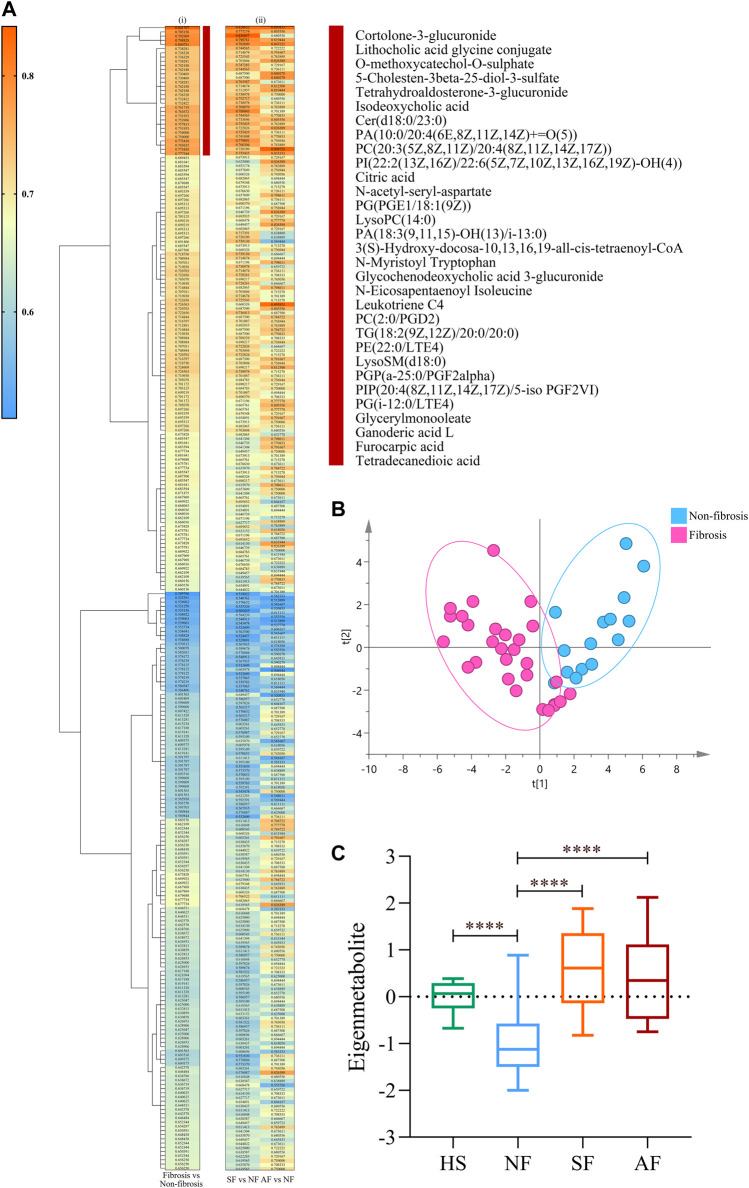
Identification of fibrosis-related metabolite fingerprint. **(Ai)** Hierarchical cluster analysis of the area under the receiver operating characteristic curve (AUC) and *p*-value differentiation between fibrosis (SF + AF) and nonsignificant fibrosis groups. Vertical brownish-red bar identified the 31-metabolite cluster, which acted as the fibrosis-associated metabolite fingerprint. **(Aii)** Corresponding metabolite AUC and *p*-value in accessing the SF or AF groups relative to fibrosis. **(B)** Principal component analysis of the fibrosis metabolite fingerprint between fibrosis and nonsignificant fibrosis DILI patients. **(C)** Change of the fibrosis eigenmetabolite in patients with different stages of fibrosis progression. Data are presented as mean ± SD. ****p < 0.0001, compared with the NF group. HS, healthy subjects; NF, nonsignificant fibrosis; SF, significant fibrosis; AF, advanced fibrosis.

Hierarchical clustering and heat map analysis were performed on the AUC and related *p*-values of 273 metabolites related to significant fibrosis. We identified the highest relevant cluster comprising 31 metabolites, which were highly correlated with fibrosis (vertical brownish-red bar, Figure 2Ai). The detail information of metabolites is shown in Supplementary Table S4. Moreover, this cluster was also effective in distinguishing SF and NF groups and AF and NF groups (Figure 2Aii). Those 31 metabolites comprised a fingerprint, and the PCA analysis showed that this fingerprint could distinguish the fibrosis group from the nonsignificant fibrosis group in DILI patients (Figure 2B). The 31 metabolites were subjected to dimensional-reduction to obtain an eigenmetabolite. Compared with healthy volunteers, there were significant differences in eigenmetabolite levels in DILI patients with fibrosis. The NF group had a depressed level of eigenmetabolite than healthy subjects, which was increased in patients with significant fibrosis. Patients with significant fibrosis had an increased eigenmetabolite level than those without fibrosis, whereas there were no statistically significant differences between SF and AF groups (Figure 2C).

**FIGURE 3:**
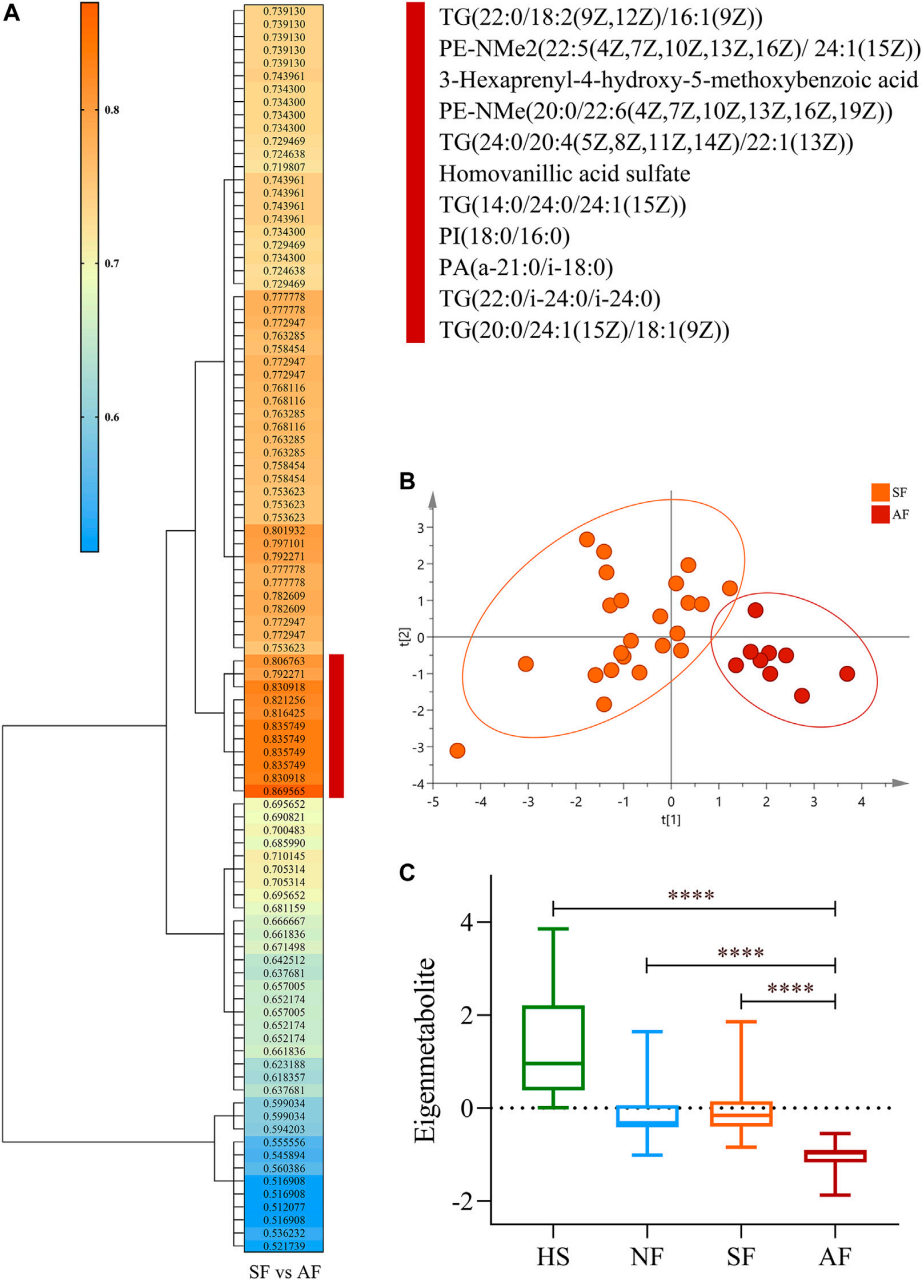
Identification of the metabolite fingerprint related to fibrosis progression. **(A)** Hierarchical cluster analysis of the area under the receiver operating characteristic curve and *p*-value differentiation between SF and AF groups. Vertical brownish-red bar identified the 11-metabolite cluster, which acted as the advanced fibrosis–associated metabolite fingerprint. **(B)** Principal component analysis of the advanced fibrosis metabolite fingerprint between the SF and AF groups. **(C)** Change of the advanced fibrosis eigenmetabolite in DILI patients with different stages of fibrosis progression. Data are presented as mean ± SD. ****p < 0.0001, compared with the AF group. HS, healthy subjects; NF, nonsignificant fibrosis; SF, significant fibrosis; AF, advanced fibrosis.

A unique metabolite fingerprint associated with advanced liver fibrosis among the 96 differential metabolites between SF and AF groups was refined in the same way. The highest relevant cluster comprising the 11 metabolites was defined (vertical brownish-red bar, Figure 3A). The detailed information of this cluster is presented in Supplementary Table S5. Those 11 metabolites formed a fingerprint related to advanced fibrosis. The PCA analysis showed that the fingerprint could distinguish the AF group and SF group (Figure 3B). Similarly, we computed an eigenmetabolite for 11-metabolite clusters. The levels of eigenmetabolite in DILI patient groups were significantly lower than those of healthy subjects. Particularly, the eigenmetabolite level in the AF group had significantly decreased. (Figure 3C).

### 3.4 Metabolite Fingerprints Reveal Progressions Over Metabolite Pathways Connected With Fibrosis in Drug-Induced Liver Injury Patients

**FIGURE 4:**
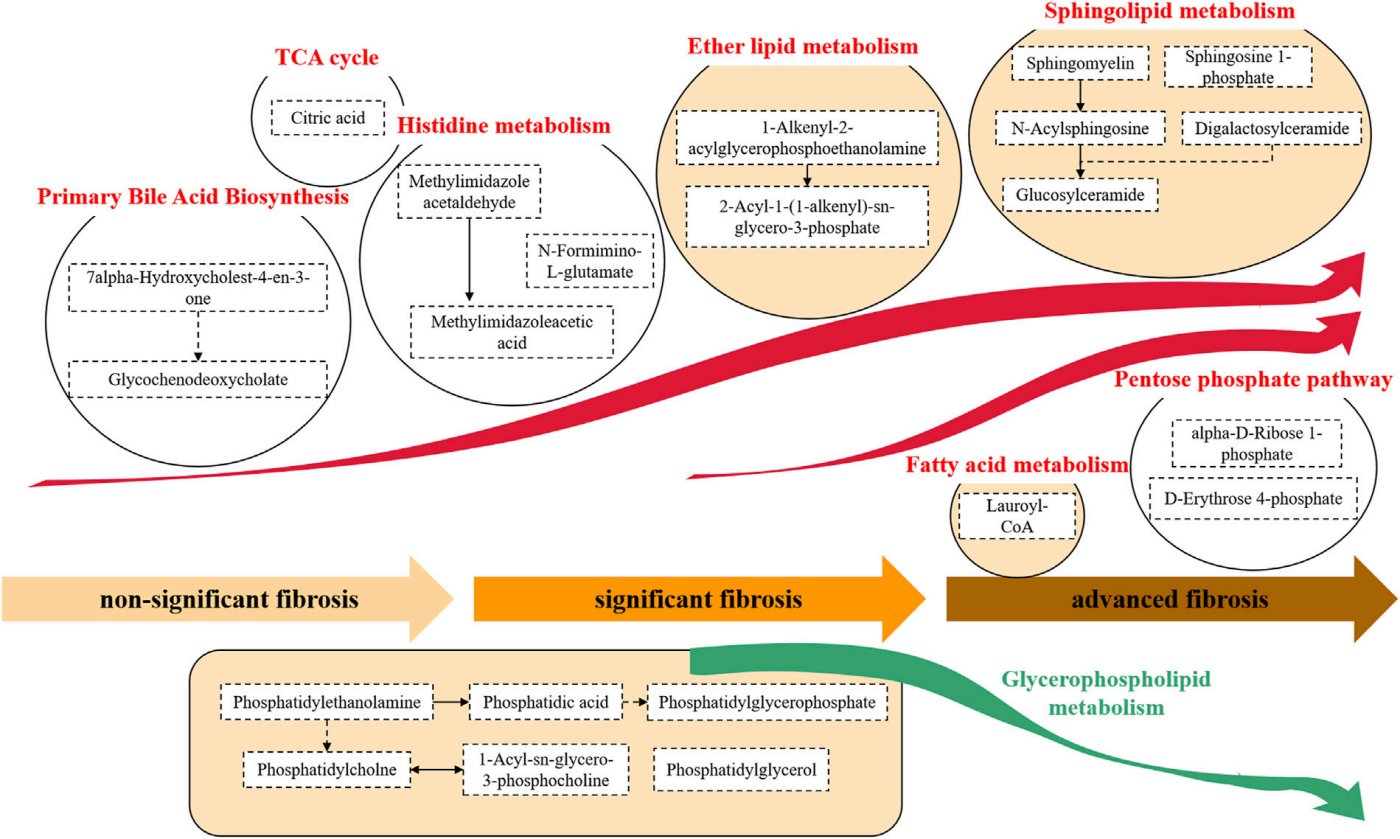
Metabolite pathway alterations involved in chronic DILI with fibrosis. A typical feature was that the synthesis of primary bile acids and histidine metabolites, and a series of metabolites related to lipids (e.g., ether lipid and sphingolipid) were significantly increased. This accumulation of lipid metabolisms persisted from the development of nonsignificant fibrosis to significant fibrosis to advanced fibrosis. In advanced fibrosis, metabolites of the fatty acid and pentose phosphate pathways were accumulated while the glycerophospholipid metabolites were found to decrease.

To understand the evolution of fibrosis in chronic DILI, two metabolite fingerprints and the associated metabolic pathway changes were identified, as shown in the schematic diagram (Figure 4).

#### 3.4.1 Metabolite Fingerprint Reveals Metabolites Associated With Primary Bile Acid Biosynthesis During the Occurrence of Drug-Induced Liver Injury–Related Fibrosis

The alterations of the metabolites in the primary bile acid biosynthesis pathway were observed in chronic DILI patients with fibrosis. We found the accumulation of the metabolites of primary bile acid biosynthesis, such as 7alpha-hydroxycholest-4-en-3-one and glycochenodeoxycholate (Figure 4).

#### 3.4.2 Metabolite Fingerprint Reveals Lipid Metabolism During the Development of Drug-Induced Liver Injury–Related Fibrosis

We observed a considerable disorder in lipid metabolites during the development of DILI-related fibrosis. A variety of lipid metabolic pathways were disrupted, including glycerophospholipid metabolism (e.g., phosphatidylethanolamine, phosphatidic acid, and phosphatidylglycerol), ether lipid metabolism [e.g., 1-alkenyl-2-acylglycerophosphoethanolamine and 2-acyl-1-(1-alkenyl)-sn- glycero-3-phosphate], sphingolipid metabolism (e.g., sphingomyelin, sphingosine 1-phosphate, and glucosylceramide), and fatty acid metabolism (e.g., lauroyl-CoA). The hyperactivity of the lipid metabolism pathway continued throughout the progression from nonsignificant fibrosis to significant fibrosis to advanced fibrosis (Figure 4).

#### 3.4.3 Metabolite Fingerprint Reveals Histidine Metabolism During the Occurrence of Drug-Induced Liver Injury–Related Fibrosis

The fibrosis-associated fingerprint revealed the metabolites in the histidine metabolic pathway, with the elevation of metabolites such as N-formimino-L-glutamate, methylimidazole acetaldehyde, and methylimidazoleacetic acid in DILI patients with fibrosis (Figure 4).

#### 3.4.4 Metabolite Fingerprint Reveals Citric Acid Elevation During the Development of Drug-Induced Liver Injury–Related Fibrosis

Citric acid, one of the components of the fibrosis-related metabolite fingerprint, was found to be elevated in DILI patients with fibrosis. Citric acid plays an important role in the tricarboxylic acid (TCA) cycle (Figure 4).

#### 3.4.5 Metabolite Fingerprint Reveals Metabolites Associated With the Pentose Phosphate Pathway During the Development of Drug-Induced Liver Injury–Related Fibrosis

We found a significant increase in the pentose phosphate–related metabolites (e.g., alpha-D-ribose-1-phosphate and D-erythrose-4-phosphate) in DILI patients with advanced fibrosis (Figure 4).

### 3.5 Metabolite Fingerprints Have a Potential Clinical Diagnostic and Prognostic Significance in Chronic Drug-Induced Liver Injury Patients With Fibrosis

**FIGURE 5:**
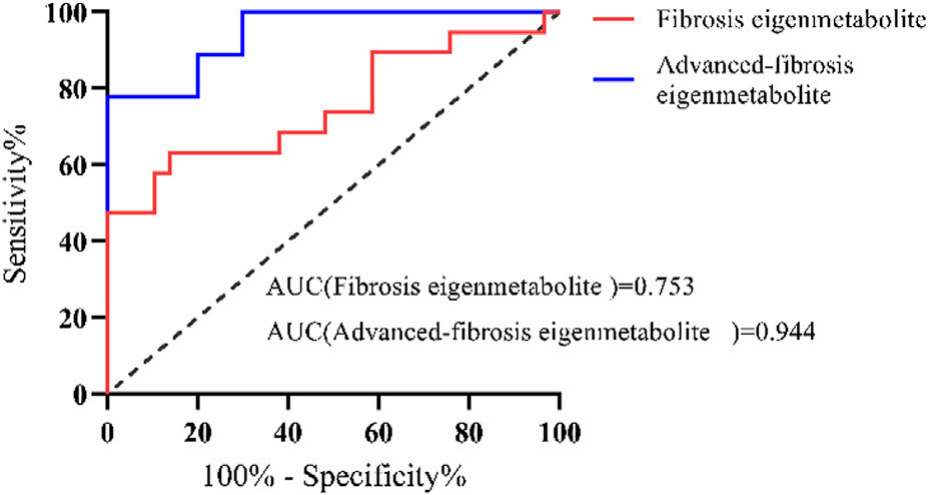
Validation of metabolite fingerprints ROC curves of the metabolite fingerprint (eigenmetabolite) associated with fibrosis and associated with advanced fibrosis in the validation group of DILI patients.

The identified metabolite fingerprints were validated in the validation group of DILI patients, and the ROC curves of metabolite fingerprints are shown in Figure 5. The results showed that both metabolite fingerprints could distinguish different stages of fibrosis in chronic DILI patients, with the AUC values 0.753 and 0.944.

**FIGURE 6:**
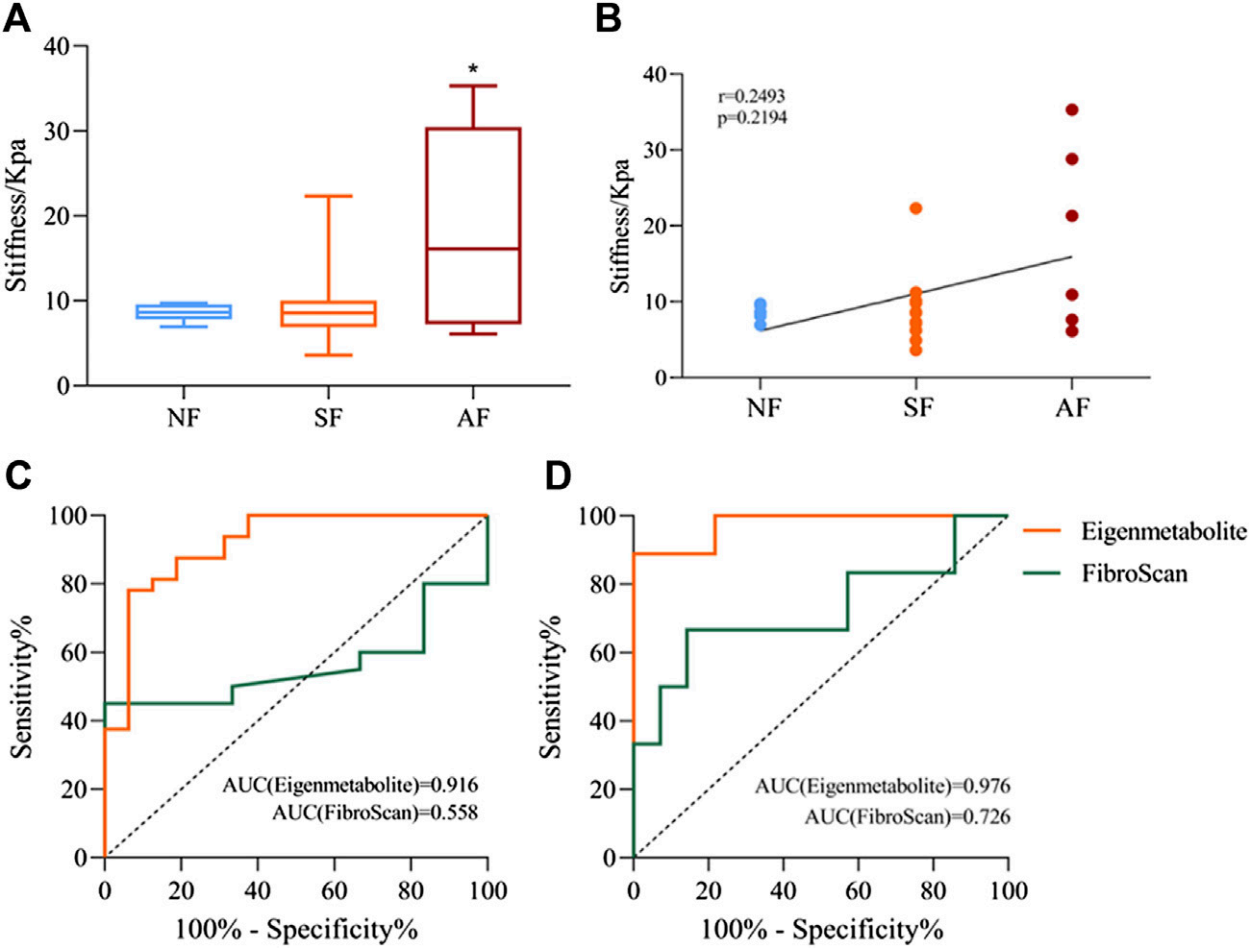
Comparison of metabolite fingerprints and FibroScan in the identification of liver fibrosis grade. **(A)** Liver stiffness values measured by FibroScan in NF, SF, and AF groups. **(B)** Spearman’s correlation analysis between liver stiffness and fibrosis grade, with the r and *p* values indicated. **(C)** Receiver operating characteristic (ROC) curves of metabolite fingerprint (eigenmetabolite) and FibroScan for the diagnosis of fibrosis and nonsignificant fibrosis patients with chronic DILI. **(D)** ROC curves of the metabolite fingerprint (eigenmetabolite) and FibroScan for the diagnosis of advanced fibrosis from significant patients with chronic DILI. Data are presented as mean ± SD. *p < 0.05, compared with the NF group. NF, nonsignificant fibrosis; SF, significant fibrosis; AF, advanced fibrosis.

We compared the metabolite fingerprints and FibroScan in the identification of liver fibrosis status. Table 2 shows the distribution of fibrosis estimated by biopsy and FibroScan. Liver stiffness was significantly increased in patients with advanced fibrosis measured by FibroScan, but there was no significant difference between the SF and AF groups (Figure 6A). Figure 6B shows the correlation between stiffness and fibrosis grade in the study of chronic DILI patients. To determine whether using metabolite fingerprinting to distinguish grades of fibrosis was clinically reliable, the ROC curves of metabolite fingerprints and FibroScan were analyzed and compared. The results showed that FibroScan had poor ability to identify the early stage of fibrosis in DILI patients (AUC = 0.558, Figure 5C) but had a better ability to distinguish patients with advanced fibrosis (AUC = 0.726, Figure 5D), while metabolite fingerprints had a good ability to identify early fibrosis (AUC = 0.916) and late fibrosis (AUC = 0.976) in DILI patients (Figures 6C,D).

**TABLE 2:** Distribution of fibrosis according to biopsy and as estimated using FibroScan.

Fibrosis according to FibroScan (LSM)	Fibrosis according to biopsy (Scheuer scores)		
	NF, S0-1 (*n* = 6)	SF, ≥S2 (*n* = 14)	AF, ≥S3 (*n* = 6)
NF (<9.4 kPa, *n* = 15)	4	9	2
SF (≥9.4 kPa, *n* = 7)	2	4	1
AF (≥12.4 kPa, *n* = 4)	0	1	3

NF, nonsignificant fibrosis; SF, significant fibrosis; AF, advanced fibrosis.

## 4 Discussion

This work described a comprehensive metabolomic evaluation in a prospective cohort of chronic DILI patients. The metabolite profile revealed significant pattern differences among NF, SF, and AF. There were 31 and 11 metabolites altered between NF and SF and SF and AF, respectively, which suggested that DILI progression might be extremely relevant to metabolic disturbance. Primary bile acid biosynthesis, histidine metabolism, and pentose phosphate pathway increased during all DILI progression, but lipid metabolism increased during nonsignificant fibrosis and significant fibrosis progression and declined during significant fibrosis and advanced fibrosis progression. Metabolite fingerprints offered excellent predictive values for distinguishing the DILI stage.

So far, liver biopsy interpretations remain the “gold standard” for the diagnosis of liver fibrosis because they are considered reliable particularly for staging fibrosis (Brunt, 2020). Liver histopathological sections were used as grouping criteria to ensure accuracy of classification. In the cases we included in the study cohort, there were no significant differences in serological measures other than albumin reduction. Albumin is a small-molecule protein produced by the liver. Patients with liver fibrosis have damaged liver cells, reduced liver function, and reduced ability to synthesize albumin. There was also a significant increase in total bilirubin and direct bilirubin in the validation cohort (Supplementary Table S3). Only a few serum indicators can be used as hints of liver lesions but cannot indicate the degree of liver fibrosis in patients.

The occurrence and progression mechanism of liver fibrosis is complex and different in various liver diseases. We found that the metabolite glycochenodeoxycholate (GCDCA) associated with bile acid synthesis was significantly increased in chronic DILI patients with fibrosis. According to reports, GCDCA has been validated to promote liver fibrosis in mice with hepatocellular cholestasis. In cholestatic mice, increased expression of αSMA and collagen1a mRNA and excess hepatic collagen deposition indicated development of liver fibrosis only after GCDCA supplementation. Bile salts may have direct pro-fibrotic effects on HSC, putatively involving EGFR and MEK1/2 signaling (Hohenester et al., 2020). In our study on chronic DILI, GCDCA was also selected as a biomarker related to fibrosis. It suggests that the mechanism of fibrosis in chronic DILI may be similar to that of cholestasis or share a part of them.

The disorder of lipid metabolism is particularly remarkable in our study. Disordered lipid metabolism has been reported as the potential biomarkers for hepatocellular, mixed, and cholestatic-type DILI, resulting in hepatocyte dysfunction and liver disease progression (Beyer, 1990; Saito et al., 2020). The levels of lysoPCs (important members of glycerophospholipid metabolism.) were markedly decreased in CCl_4_-induced hepatic fibrosis mice (Zhang et al., 2021). Sphingolipids were structural components of biological membranes, and many of them, such as sphingosine 1-phosphate (S1P), are also now recognized to be important mediators of many basic cellular processes involved in tissue responses to injury (Rivera and Chun, 2008). Numerous studies have suggested important roles for S1P in the development of fibrosis in the lungs, skin, liver, heart, and eye, and alterations in circulating S1P levels have been observed in various human diseases characterized by tissue fibrosis (Shea and Tager, 2012), which is consistent with our results. Interestingly, in our study, the trend of lipid metabolism associated with fibrosis in DILI was not constant. It did not decrease at the beginning of fibrosis but decreased sharply at the advanced stage. This is because of the unique mechanism of DILI. At the same time, we observed a significant increase of the metabolite, citric acid, in patients with chronic DILI–related fibrosis. The correlation between citric acid and lipid metabolism has been studied in alcoholic fatty liver. The TCA cycle mainly occurs in mitochondria, which is the core of intermediate metabolism. Progressive alteration of the mitochondria decreases fatty acid oxidation by interfering with citric acid cycle activity (Baraona and Lieber, 1979).

We found an abnormal increase in histidine metabolism in DILI patients with fibrosis. Histidine phosphorylation may mediate in TGF-β1–induced HSC activation and CCl_4_-induced liver fibrosis (Gong et al., 2020). Abnormalities in the pentose phosphate pathway (PPP) had also been observed in DILI patients with advanced fibrosis. Metabolism of glucose through the PPP provides a variety of raw materials for anabolism, such as ribose 5-phosphate and NADPH. It had been reported that liver cirrhosis patients suffer from complete deficiency of PPP enzyme transaldolase (Perl, 2007; Lipinski et al., 2018).

DILI as a challenging liver disorder has never had a specific method for its diagnosis because it can present with a range of phenotypes, mimicking almost every other hepatic disease. The absence of diagnostic DILI biomarkers has led to that serum AST/ALT, ALP, and TBL levels still remain the pillars for DILI case detection and qualification (Andrade and Robles-Díaz, 2020). FibroScan is recommended as a non-invasive tool to assess fibrosis according to the European Association for the Study of the Liver guidelines and World Health Organization guidelines (Huang et al., 2021b). However, the accuracy of FibroScan in chronic DILI patients has been discussed rarely. In our study, we found that FibroScan’s ability to distinguish NF from SF is not as effective as expected in chronic DILI patients. This is consistent with previous reports regarding the discordance in fibrosis staging between liver biopsy and FibroScan in obese patients with chronic liver disease (Myers et al., 2012). A pilot study clearly indicates that transient elastography cannot correctly classify all patients with persistent cirrhosis following 4 years of a patient with a sustained virological response (SVR) (D'Ambrosio et al., 2013). All of the presence of inflammation and obesity or ascites can compromise the diagnostic accuracy of FibroScan (Myers et al., 2012; Lupsor-Platon and Badea, 2015; Huang et al., 2021b). Of course, there are several limitations in our study. The first is that the diagnostic accuracy of biopsy was limited by sampling error and inter- and intra-observer variability. The second is a limited number of patients enrolled in this study. In spite of this, it is gratifying to see the great potential of serum metabolite fingerprints in distinguishing the degree of fibrosis in our study.

In conclusion, we reported a comprehensive metabolomic evaluation for identifying clinically relevant perturbations in circulating metabolites in chronic DILI. The bile acid synthesis pathway, lipid metabolism pathway, pentose phosphate pathway, and histidine metabolism pathway might be potential target pathways for the treatment of chronic liver diseases. This evaluation provided a new understanding of chronic DILI pathogenesis and a potential strategy for therapeutic intervention. Those biomarker panels showed a satisfactory diagnostic performance with regard to the receiver operating characteristic curves, suggesting that the differential metabolites have a very potential diagnostic method for the staging of liver fibrosis in chronic DILI patients.

## Data Availability

The original contributions presented in the study are included in the article/Supplementary Material, further inquiries can be directed to the corresponding authors.
