# Supplementary material for: Metabolomic Profiling for Histologically Fibrotic Stage in Chronic Drug-Induced Liver Injury
Source: Front Pharmacol. 2022 May 20;13:896198. doi: 10.3389/fphar.2022.896198 (PMC9163384; doi:10.3389/fphar.2022.896198)
Supplement: Supplementary file 1 [file DataSheet1.docx]

**Supplementary Table1**

**A** Pathological diagnostic criteria of chronic hepatitis

| classification | Degree of inflammation | |
| --- | --- | --- |
|  | The portal area and surrounding | hepatic lobules |
| G0 | no inflammation present | no inflammation present |
| G1 | Inflammation in the portal area | Degeneration and a few necrotic lesions |
| G2 | Mild fragmentary necrosis | Degeneration, spot, focal necrosis or eosinophilic bodies |
| G3 | Moderate fragmentary necrosis | Severe deformation, necrosis or bridging necrosis |
| G4 | Severe fragmentary necrosis | Extensive bridging necrosis, involving multiple lobules, lobule structure disorder (polylobule necrosis) |

**B** The detailed histopathological diagnosis in DILI patients

| Patients number | Pathological diagnosis | | Hepatocellular injury type | Fibrosis group |
| --- | --- | --- | --- | --- |
|  | Grades of chronic hepatitis | hepatic fibrosis |  |  |
| 100175513 | G2-3 | the portal area expanded | mixed type | NF |
| 3001181309 | G3 | the portal area expanded | hepatocellular injury type | NF |
| 3001197075 | G3 | the portal area expanded | mixed type | NF |
| 3001209790 | G1 | the portal area expanded | mixed type | NF |
| 3001240475 | G1 | the portal area expanded | cholestasis type | NF |
| 3000941428 | G1 | the portal area expanded | cholestasis type | NF |
| 3001053781 | G1 | no fibrosis | cholestasis type | NF |
| 3001065461 | G1 | no fibrosis | cholestasis type | NF |
| 3001117567 | G2 | the portal area expanded | cholestasis type | NF |
| 3001117753 | G1 | the portal area expanded | cholestasis type | NF |
| 3001119634 | G1 | the portal area expanded | cholestasis type | NF |
| 3001124893 | G2 | the portal area expanded | hepatocellular injury type | NF |
| 3001126523 | G1 | the portal area expanded | cholestasis type | NF |
| 3001141727 | G1 | the portal area expanded | cholestasis type | NF |
| 3001149665 | G1 | no fibrosis | cholestasis type | NF |
| 3000999006 | G1 | no fibrosis | mixed type | NF |
| 3001099759 | G3 | the portal area was fibrotic and fibrous septa formed | hepatocellular injury type | SF |
| 3001124369 | G2 | the portal area was fibrotic and fibrous septa formed | cholestasis type | SF |
| 3001167914 | G3 | the portal area was fibrotic and fibrous septa formed | hepatocellular injury type | SF |
| 3001202722 | G2 | the portal area was fibrotic and fibrous septa formed- | cholestasis type | SF |
| 3001224486 | G3 | the portal area expanded | hepatocellular injury type | SF |
| 3001230818 | G3 | the portal area was fibrotic and fibrous septa formed | mixed type | SF |
| 3001231149 | G3 | the portal area was fibrotic and fibrous septa formed | mixed type | SF |
| 3001234500 | G3-4 | the portal area was fibrotic and fibrous septa formed | hepatocellular injury type | SF |
| 100175164 | G3 | the portal area expanded | hepatocellular injury type | SF |
| 3000076291 | G2 | the portal area expanded | hepatocellular injury type | SF |
| 3000898857 | G2 | the portal area was fibrotic and fibrous septa formed | cholestasis type | SF |
| 3000959987 | G3 | the portal area was fibrotic and fibrous septa formed | mixed type | SF |
| 3001011036 | G3 | the portal area was fibrotic and fibrous septa formed | mixed type | SF |
| 3001045638 | G2 | the portal area was fibrotic and fibrous septa formed | cholestasis type | SF |
| 3001065068 | G3 | the portal area expanded | hepatocellular injury type | SF |
| 3001065473 | G2 | the portal area was fibrotic and fibrous septa formed | hepatocellular injury type | SF |
| 3001098206 | G3 | the portal area was fibrotic and fibrous septa formed | hepatocellular injury type | SF |
| 3001133071 | G4 | the portal area was fibrotic and fibrous septa formed | mixed type | SF |
| 3001136821 | G3 | the portal area was fibrotic and fibrous septa formed | hepatocellular injury type | SF |
| 3001166082 | G3 | the portal area was fibrotic and fibrous septa formed | hepatocellular injury type | SF |
| 3000898272 | G2 | the portal area was fibrotic and fibrous septa formed | hepatocellular injury type | SF |
| 3001000670 | G2 | the portal area was fibrotic and fibrous septa formed | hepatocellular injury type | SF |
| 3001006209 | G2 | the portal area expanded | hepatocellular injury type | SF |
| 3001215875 | G3 | fibrous septa accompanied by disordered lobules | hepatocellular injury type | AF |
| 3001227573 | G2 | possible or certain cirrhosis | mixed type | AF |
| 3001239089 | G2 | fibrous septa accompanied by disordered lobules | hepatocellular injury type | AF |
| 3001024677 | G2 | fibrous septa accompanied by disordered lobules | cholestasis type | AF |
| 3001055637 | G3-4 | possible or certain cirrhosis | mixed type | AF |
| 3001099760 | G3 | fibrous septa accompanied by disordered lobules | hepatocellular injury type | AF |
| 3001134480 | G4 | fibrous septa accompanied by disordered lobules | mixed type | AF |
| 3001146272 | G2 | fibrous septa accompanied by disordered lobules | cholestasis type | AF |
| 3000990066 | G3-4 | fibrous septa accompanied by disordered lobules | hepatocellular injury type | AF |

**Supplementary Table2|** Parameter settings for LC–MS/MS

**A**

| **Time(min)** | **A(%)** | **B(%）** |
| --- | --- | --- |
| 0 | 95 | 5 |
| 1 | 95 | 5 |
| 9 | 60 | 40 |
| 19 | 10 | 90 |
| 21 | 0 | 100 |
| 25 | 0 | 100 |

**B**

|  | **Positive** | **Negative** |
| --- | --- | --- |
| **Capillary voltage(kV)** | 2.5 ESI+ | 2.2 ESI- |
| **Cone voltage(V)** | 40 | |
| **Ion source temperature (◦C)** | 130 | |
| **Desolvation temperature(◦C)** | 350 | |
| **Cone Airflow(L/h）** | 50 | |
| **Desolvation airflow (L/h）** | 800 | |
| **mass range(m/z)** | 50 ~ 1,200 | |

**Supplementary Table3|** Clinical characteristics of the validation patients in NF, SF, and AF groups.

| **Characteristics** | **NF(n=29)** | **SF(n=10)** | **AF(n=9)** | ***P*-value** | | |
| --- | --- | --- | --- | --- | --- | --- |
|  |  |  |  | **NF *vs* SF** | **NF *vs* AF** | **SF *vs* AF** |
| Age/year | 47.0(38.0,57.0) | 48.0(46.0,51.8) | 52.0(44.0,59.0) | 0.537 | 0.297 | 0.709 |
| Female, n (%) | 24(82.7) | 8(80.0) | 7(77.7) | - | - | - |
| ALT/U·L−1 | 23.0(16.0,48.0) | 43.0(29.5,91.3) | 41.0(34,118) | 0.210 | 0.260 | 0.888 |
| AST/U·L−1 | 27.0(20.0,52.0) | 39.5(31,122.75) | 112(33,151) | 0.442 | 0.074 | 0.392 |
| AST/ALT | 1.3(0.9,1.7) | 1.1(0.9,1.2) | 1.2(1.1,2.8) | 0.474 | 0.873 | 0.100 |
| ALP/U·L−1 | 88.0(70.0,112.0) | 109.5(95.5,147.5) | 115.0(97.5,163.0) | 0.152 | 0.148 | 0.977 |
| GGT/U·L−1 | 49.0(17.0,124.0) | 66.0(39.3,89.3) | 98.0(62.0,231.0) | 0.955 | 0.051 | 0.179 |
| ALB/g·L−1 | 39.0(37.0,40.0) | 36.5(35.3,38.0) | 32.0(26.0,39.0) | 0.051 | 0.000 | 0.078 |
| TBIL/µmo·L−1 | 10.6(8.4,13.7) | 14.9(10.8,21.0) | 54.9(23.0,62.7) | 0.171 | 0.000 | 0.014 |
| DBIL/µmo·L−1 | 3.6(2.7,4.8) | 5.3(4.4,10.7) | 37.6(14.2,49.4) | 0.236 | 0.000 | 0.018 |
| CHE/U·L−1 | 6633.5(5570.0,7041.8) | 5342.0(4728.0,5946.0) | 5402.0(4831.3,5862.0) | 0.065 | 0.095 | 0.819 |
| TC/mmol·L−1 | 4.3(3.7,5.1) | 3.7(3.2,4.3) | 4.5(3.3,5.5) | 0.099 | 0.097 | 0.203 |
| TG/mmol·L-1 | 1.2(1.0,2.0) | 1.2(1.0,1.4) | 1.4(1.1,1.5) | 0.215 | 0.757 | 0.216 |
| IgA/g·L−1 | 2.2(1.8,2.8) | 2.6(1.9,3.2) | 3.1(2.7,3.9) | 0.285 | 0.111 | 0.655 |
| IgG/g·L−1 | 12.6(10.5,13.4) | 13.4(9.2,14.9) | 16.2(14.1,17.1) | 0.668 | 0.186 | 0.088 |
| IgM/g·L−1 | 1.4(0.8,1.8) | 1.2(0.7,1.8) | 1.6(1.1,2.2) | 0.456 | 0.346 | 0.267 |
| INR/IU | 0.9(0.9,1.0) | 1.0(0.9,1.0) | 1.01.0,1.2) | 0.123 | 0.167 | 0.051 |
| CRE/µmo·L−1 | 62.0(58.0,69.0) | 63.5(60.0,70.5) | 61.0(54.0,67.0) | 0577 | 0.884 | 0.585 |
| PT/s | 10.6(9.9,11.0) | 11.1(10.7,11.9) | 11.6(11.1,12.5) | 0.119 | 0.052 | 0.549 |

Data are median (p25, p75) or numerical value. P-values for comparisons were carried out by nonparametric tests.
Abbreviations: ALT, alanine aminotransferase; AST, aspartate transaminase; ALP, alkaline phosphatase; GGT, gamma-glutamyl transpeptidase; ALB, albumin; TBIL, total bilirubin; DBIL, direct bilirubin; CHE, Cholinesterase; TC, total cholesterol; TG, triglyceride; INR, International standard ratio ; CRE, creatinine; PT, prothrombin time.

**Supplementary Table4 |** Details of the metabolite fingerprint associated with DILI fibrosis

| **NO.** | **Ion Mode** | ***m/z*** | **RT** | **HMDB ID** | **Formula** | **Common Name** | **Fold Change** | **Log2(FC)** | **AUC** | ***p*-value** |  |
| --- | --- | --- | --- | --- | --- | --- | --- | --- | --- | --- | --- |
|  |  |  |  |  |  |  |  |  |  |  |  |
| 1 | ESI- | 541.2627 | 4.24min | HMDB0010320 | C27H42O11 | Cortolone-3-glucuronide | 3.4973 | 1.8063 | 0.8418 | 0.0000 |  |
| 2 | ESI+ | 434.3164 | 8.27min | HMDB0000698 | C26H43NO4 | Lithocholic acid glycine conjugate | 0.3142 | -1.6702 | 0.7852 | 0.0105 |  |
| 3 | ESI- | 203.0001 | 1.20min | HMDB0060013 | C7H8O5S | O-methoxycatechol-O-sulphate | 0.4195 | -1.2533 | 0.7930 | 0.0092 |  |
| 4 | ESI- | 481.3107 | 15.05min | HMDB0259391 | C27H46O5S | 5-Cholesten-3beta-25-diol-3-sulfate | 1.5896 | 0.6686 | 0.7988 | 0.0006 |  |
| 5 | ESI- | 539.2481 | 4.45min | HMDB0010357 | C27H40O11 | Tetrahydroaldosterone-3-glucuronide | 2.1892 | 1.1304 | 0.8008 | 0.0001 |  |
| 6 | ESI- | 391.2837 | 16.27min | HMDB0002536 | C24H40O4 | Isodeoxycholic acid | 0.5307 | -0.9142 | 0.7383 | 0.0378 |  |
| 7 | ESI+ | 638.652 | 24.25min | HMDB0011767 | C41H83NO3 | Cer(d18:0/23:0) | 2.4280 | 1.2798 | 0.7363 | 0.0222 |  |
| 8 | ESI- | 625.3413 | 4.61min | HMDB0262689 | C33H55O9P | PA(10:0/20:4(6E,8Z,11Z,14Z)+=O(5)) | 0.3978 | -1.3300 | 0.7363 | 0.0180 |  |
| 9 | ESI+ | 832.5719 | 16.80min | HMDB0008379 | C48H82NO8P | PC(20:3(5Z,8Z,11Z)/20:4(8Z,11Z,14Z,17Z)) | 0.3826 | -1.3861 | 0.7383 | 0.0163 |  |
| 10 | ESI+ | 979.5886 | 8.22min | HMDB0277767 | C53H87O14P | PI(22:2(13Z,16Z)/22:6(5Z,7Z,10Z,13Z,16Z,19Z)-OH(4)) | 0.2768 | -1.8532 | 0.7422 | 0.0211 |  |
| 11 | ESI+ | 193.0394 | 1.32min | HMDB0000094 | C6H8O7 | Citric acid | 1.8351 | 0.8759 | 0.7422 | 0.0197 |  |
| 12 | ESI- | 261.0738 | 3.75min | HMDB0062501 | C9H14N2O7 | N-acetyl-seryl-aspartate | 4.5669 | 2.1912 | 0.7305 | 0.0252 |  |
| 13 | ESI+ | 847.5155 | 6.95min | HMDB0269064 | C44H79O13P | PG(PGE1/18:1(9Z)) | 1.5952 | 0.6737 | 0.7305 | 0.0271 |  |
| 14 | ESI+ | 468.2184 | 8.22min | HMDB0010379 | C22H30FN3O7 | LysoPC(14:0) | 0.4142 | -1.2717 | 0.7383 | 0.0092 |  |
| 15 | ESI- | 643.4004 | 21.87min | HMDB0267506 | C34H61O9P | PA(18:3(9,11,15)-OH(13)/i-13:0) | 1.7764 | 0.8290 | 0.7422 | 0.0035 |  |
| 16 | ESI+ | 1098.353 | 21.32min | HMDB0060210 | C43H70N7O18P3S | 3(S)-Hydroxy-docosa-10,13,16,19-all-cis-tetraenoyl-CoA | 3.7245 | 1.8971 | 0.7422 | 0.0074 |  |
| 17 | ESI+ | 415.3035 | 8.22min | HMDB0242061 | C25H38N2O3 | N-Myristoyl Tryptophan | 0.2358 | -2.0846 | 0.7363 | 0.0043 |  |
| 18 | ESI+ | 626.3515 | 8.52min | HMDB0002579 | C32H51NO11 | Glycochenodeoxycholic acid 3-glucuronide | 0.3631 | -1.4615 | 0.7324 | 0.0059 |  |
| 19 | ESI+ | 416.3065 | 8.22min | HMDB0242073 | C26H41NO3 | N-Eicosapentaenoyl Isoleucine | 0.2654 | -1.9139 | 0.7324 | 0.0032 |  |
| 20 | ESI- | 624.3374 | 4.59min | HMDB0001198 | C30H47N3O9S | Leukotriene C4 | 0.3862 | -1.3724 | 0.7617 | 0.0158 |  |
| 21 | ESI+ | 634.3461 | 8.34min | HMDB0288828 | C30H52NO11P | PC(2:0/PGD2) | 0.3718 | -1.4275 | 0.7637 | 0.0220 |  |
| 22 | ESI+ | 943.8556 | 24.58min | HMDB0005467 | C61H114O6 | TG(18:2(9Z,12Z)/20:0/20:0) | 2.1541 | 1.1071 | 0.7520 | 0.0152 |  |
| 23 | ESI+ | 959.5915 | 9.60min | HMDB0283748 | C50H91N2O11PS | PE(22:0/LTE4) | 0.2760 | -1.8572 | 0.7539 | 0.0156 |  |
| 24 | ESI- | 465.3566 | 18.10min | HMDB0012082 | C23H51N2O5P | LysoSM(d18:0) | 0.4604 | -1.1190 | 0.7578 | 0.0048 |  |
| 25 | ESI- | 1025.632 | 15.06min | HMDB0274711 | C51H96O16P2 | PGP(a-25:0/PGF2alpha) | 1.6579 | 0.7294 | 0.7520 | 0.0050 |  |
| 26 | ESI+ | 1009.451 | 22.16min | HMDB0280120 | C47H78O19P2 | PIP(20:4(8Z,11Z,14Z,17Z)/5-iso PGF2VI) | 2.6946 | 1.4301 | 0.7500 | 0.0013 |  |
| 27 | ESI+ | 850.4745 | 13.87min | HMDB0271083 | C41H72NO13PS | PG(i-12:0/LTE4) | 2.1664 | 1.1153 | 0.7500 | 0.0021 |  |
| 28 | ESI+ | 371.2903 | 13.27min | HMDB0252857 | C21H38O5 | Glycerylmonooleate | 0.2012 | -2.3131 | 0.7734 | 0.0202 |  |
| 29 | ESI- | 533.2981 | 15.06min | HMDB0035885 | C30H46O8 | Ganoderic acid L | 1.5742 | 0.6547 | 0.7656 | 0.0022 |  |
| 30 | ESI+ | 295.2237 | 1.44min | HMDB0112099 | C18H30O3 | Furocarpic acid | 2.1636 | 1.1134 | 0.7734 | 0.0084 |  |
| 31 | ESI- | 257.1748 | 6.05min | HMDB0000872 | C14H26O4 | Tetradecanedioic acid | 0.1742 | -2.5208 | 0.7773 | 0.0066 |  |

ESI- represents negative ion mode with [M-H] adduct type; ESI+ represents positive ion mode with [M+H] adduct type.

**Supplementary Table5** | Details of the metabolite fingerprint associated with DILI advanced-fibrosis

| **NO.** | **Ion**  **Mode** | ***m/z*** | **RT** | **HMDB ID** | **Formula** | **Common Name** | **Fold Change** | **Log2(FC)** | **AUC** | **P-value** |
| --- | --- | --- | --- | --- | --- | --- | --- | --- | --- | --- |
| 1 | ESI+ | 1000.786 | 24.36min | HMDB0045847 | C65H122O6 | TG(20:0/24:1(15Z)/18:1(9Z)) | 2.5625 | 0.7366 | 0.8696 | 0.0135 |
| 2 | ESI- | 261.0073 | 1.12min | HMDB0011719 | C9H10O7S | Homovanillic acid sulfate | 0.3850 | -0.7262 | 0.8357 | 0.0041 |
| 3 | ESI- | 999.7319 | 21.55min | HMDB0042255 | C67H100O6 | TG(14:0/24:0/24:1(15Z)) | 2.2362 | 0.8613 | 0.8357 | 0.0063 |
| 4 | ESI+ | 839.5741 | 21.84min | HMDB0009805 | C47H83O10P | PI(18:0/16:0) | 2.1349 | 0.9139 | 0.8357 | 0.0160 |
| 5 | ESI+ | 747.5998 | 13.21min | HMDB0115739 | C42H83O8P | PA(a-21:0/i-18:0) | 2.3590 | 0.8076 | 0.8357 | 0.0123 |
| 6 | ESI- | 575.3979 | 21.79min | HMDB0000977 | C38H56O4 | 3-Hexaprenyl-4-hydroxy-5-methoxybenzoic acid | 2.8952 | 0.6520 | 0.8309 | 0.0457 |
| 7 | ESI+ | 1116.549 | 11.55min | HMDB0069877 | C73H142O6 | TG(22:0/i-24:0/i-24:0) | 2.9045 | 0.6501 | 0.8309 | 0.0110 |
| 8 | ESI+ | 834.5984 | 15.28min | HMDB0113305 | C48H84NO8P | PE-NMe(20:0/22:6(4Z,7Z,10Z,13Z,16Z,19Z)) | 0.4290 | -0.8190 | 0.8213 | 0.0018 |
| 9 | ESI- | 1047.906 | 4.73min | HMDB0047468 | C69H124O6 | TG(24:0/20:4(5Z,8Z,11Z,14Z)/22:1(13Z)) | 0.2466 | -0.4951 | 0.8164 | 0.0023 |
| 10 | ESI+ | 913.6033 | 21.06min | HMDB0046634 | C59H108O6 | TG(22:0/18:2(9Z,12Z)/16:1(9Z)) | 2.1876 | 0.8855 | 0.8068 | 0.0491 |
| 11 | ESI+ | 904.4579 | 20.65min | HMDB0114548 | C53H94NO8P | PE(P-16:0/14:0) | 1.9428 | 1.0437 | 0.7923 | 0.0425 |

ESI- represents negative ion mode with [M-H] adduct type; ESI+ represents positive ion mode with [M+H] adduct type.
